# Diabetic retinopathy may predict the renal outcomes of patients with diabetic nephropathy

**DOI:** 10.1080/0886022X.2018.1456453

**Published:** 2018-04-10

**Authors:** Junlin Zhang, Yiting Wang, Li Li, Rui Zhang, Ruikun Guo, Hanyu Li, Qianqian Han, Geer Teng, Fang Liu

**Affiliations:** a Division of Nephrology, West China Hospital of Sichuan University, Chengdu, China;; b Faculty of Social Development & Western China Development Studies, Sichuan University, Chengdu, China

**Keywords:** Diabetic nephropathy, diabetic retinopathy, renal pathology, risk factors, renal outcomes

## Abstract

**Background:** The patients with Type 2 diabetes mellitus (T2DM) and diabetic retinopathy (DR) are prone to develop diabetic nephropathy (DN). In this study, we aimed to clarify the relationship between DR and the progression of DN in patients with T2DM.

**Methods:** In the cross-section study, 250 patients with T2DM and biopsy-proven DN were divided into two groups: 130 in the DN without DR group (DN group) and 120 in the DN + DR group. Logistic regression analysis was performed to identify risk factors for DR. Of the above 250 patients, 141 were recruited in the cohort study who received follow-up for at least 1 year and the influence of DR on renal outcome was assessed using Cox regression. Renal outcome was defined as the progression to end-stage renal disease (ESRD).

**Results:** In the cross-section study, the severity of glomerular lesions (class IIb + III) and DM history >10 years were significantly associated with the odds of DR when adjusting for baseline proteinuria, hematuria, e-GFR, and interstitial inflammation. In the cohort study, a multivariate COX analysis demonstrated that the DR remained an independent risk factor for progression to ESRD when adjusting for important clinical variables and pathological findings (*p* < .05).

**Conclusions:** These findings indicated that the severity of glomerular lesions was significantly associated with DR and DR was an independent risk factor for the renal outcomes in patients with DN, which suggested that DR may predict the renal prognosis of patients with T2DM and DN.

## 1. Introduction

Type 2 diabetes mellitus (T2DM) is now an enormous and ever-growing epidemic in the world and one of the most important public health challenges of the 21st century [1–3]. Accordingly, the prevalence of diabetic nephropathy (DN) and diabetic retinopathy (DR), two major chronic microvascular complications of T2DM patients, has increased in parallel with diabetes.

DN, characterized by persistent albuminuria, hypertension, and progressive renal failure [4–6], is now the leading cause of end-stage renal (ESRD) in developed countries and worldwide [7–9]. Moreover, a recent study indicates that diabetic kidney disease has become more common than chronic kidney disease related to glomerulonephritis in China [10]. The diagnosis of DN is, in most cases, based on the clinical manifestations, and renal biopsy is only performed in patients with atypical presentations. However, recent extensive studies suggest that the incidence of nondiabetic renal disease (NDRD, such as IgA nephropathy and membranous nephropathy) in patients with T2DM varies from 27% to 82.9% [11–13]. Consequently, the results of some studies about DN in line with the clinical symptoms were controversial.

DR is known to be the primary cause of blindness in the working-age population and can develop without any serious symptoms. Globally, the number of people with DR will grow from 126.6 million in 2010 to 191.0 million by 2030, and the number with vision-threatening diabetic retinopathy (VTDR) will increase from 37.3 to 56.3 million, if prompt action is not taken [14]. In 2016, the theme of World Diabetes Day was Eyes on Diabetes. The focus will emphasize the importance of screening to ensure early diagnosis of DR and treatment to cut down the risk of blindness. While certain risk factors for DR, like the type and duration of DM, cannot be modified, control of other convertible risk factors such as hyperglycemia, hypertension, and hyperlipidemia is efficient and crucial to reduce DR-related blindness [15]. Moreover, for nephrologists, earlier detection, early diagnosis, and prevention of DR in patients with DN should be emphasized, not just for the diagnosis of DN.

Previous studies indicated that DR was significantly associated with renal function deterioration and patients with DN experienced higher incidence of DR as compared with patients without DN [16–20]. However, the diagnosis of DN in their studies was based on the clinical manifestations and NDRD patients are potentially misdiagnosed with DN, which lead to the results were less convincing. In this report, we aimed to identify whether DR was associated with the progression of DN in patients with T2DM and biopsy-proven DN.

## 2. Materials and methods

### 2.1. Inclusion and exclusion criteria

A total of 470 in-patients with DM who received renal biopsy in West China Hospital from July 2001 to February 2017 were reviewed, 250 patients were eligible and recruited in the cross-section study. The diagnosis of T2DM was based on the criteria of the American Diabetes Association (ADA) [21]. DR was defined as present if any of the following lesions was detected: microaneurysms, retinal hemorrhages, soft exudates, hard exudates, or vitreous hemorrhage. DN was defined according to the criteria in the recent study published in 2015 by An et al. [22], and was diagnosed by at least two renal pathologists and/or nephrologists according to the Tervaert’s classification [23]. The general indications for renal biopsy in our present study were T2DM patients with renal damage (defined as the abnormal urinalysis or renal dysfunction) who were absent from absolute contraindications, especially T2DM patients without DR or T2DM patients with obvious glomerular hematuria, massive proteinuria, and/or short diabetic duration. Exclusion criteria were consisted of patients with non-T2DM, without DR examination, or the patients who had nondiabetic renal diseases such as IgA nephropathy or membranous nephropathy. The patients having accepted dialysis or kidney transplantation before renal biopsy were also excluded. This study protocol was reviewed and approved by the ethics committee of West China Hospital of Sichuan University. Written informed consent was obtained from all individual participants when hospitalized.

#### 2.1.1. Cross-section study

This DR phenotype cross-section study was performed to find the risk factors for the odds of DR in the diabetic patients with DN. All of 250 patients were divided into two groups: 130 in the DN without DR group (DN group) and 120 in the DN + DR group.

#### 2.1.2. Retrospective cohort study

**Figure 1.**
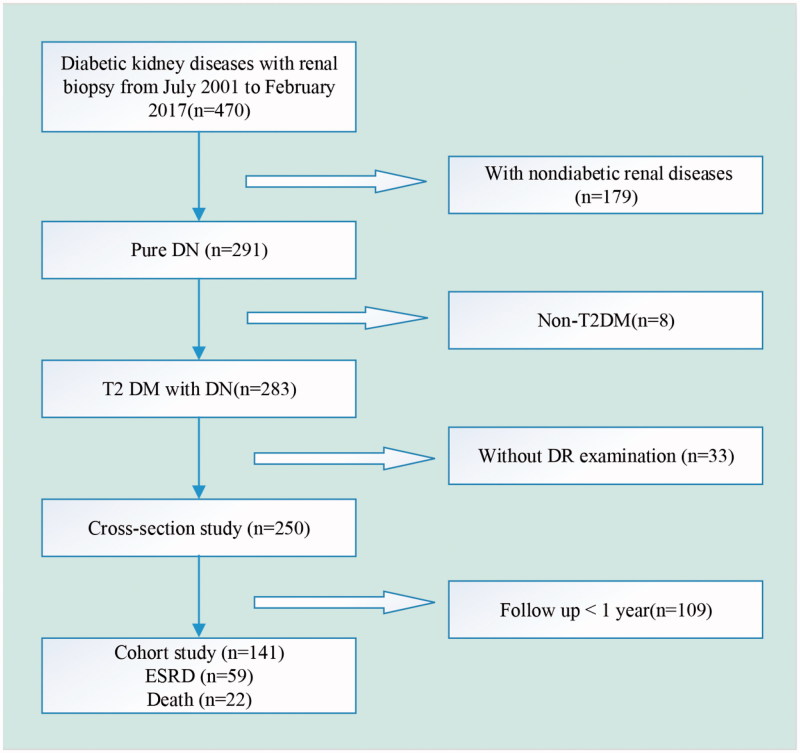
Flowchart of study participants.

The cohort study was designed to clarify whether DR further influenced renal progression and induced poorer outcomes in DN + DR group than DN group. Of the above 250 patients, 141 patients, including 73 in DN + DR group and 68 in DN group, who received follow-up for at least 1 year after kidney biopsy, were recruited (Figure 1). Renal outcome was defined by the progression to ESRD, which was e-GFR <15 mL/min/1.73 m^2^ or the initiation of renal replacement therapy, and was the end point of our study. Renal survival was assessed by using whether the patients reached the end point of our study during the follow up. Patients not reaching the end point were evaluated using the medical records of their last follow-up visit.

### 2.2. Clinical and laboratory information

The baseline clinical features, which were collected within 1 month of kidney biopsy, included age and duration of diabetes at the time of biopsy, gender, blood pressure, weight, height, fasting blood glucose, HbA1c, 24-h urinary protein, serum creatinine, and estimated glomerular filtration rate (e-GFR, calculated by the CKD-EPI formula). In the present study, proteinuria is measured from the total quantity of urine protein in 24 h. Diabetic retinopathy lesions were examined at the time of biopsy or prior to their admission by experienced ophthalmologists. In some patients, equivocal diagnosis was validated with optical coherence tomography (OCT) and fundus color photography.

### 2.3. Renal pathology

**Figure 2.**
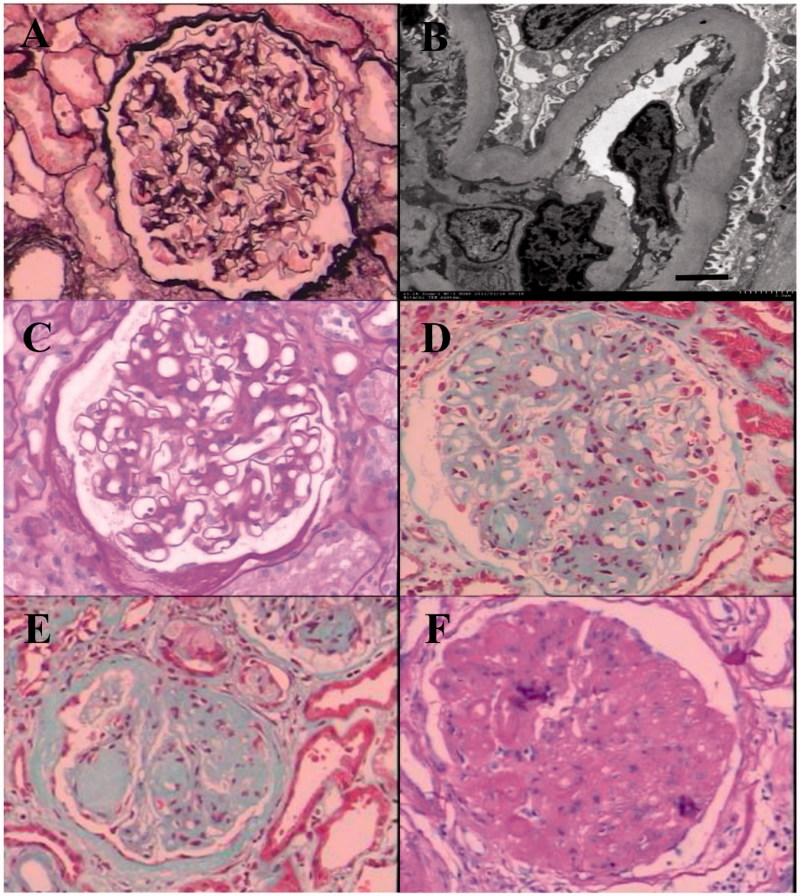
Representative examples of different glomerular classes in DN. (A) Mild changes by light microscopy (Class I). (B) GBM thickening by electron microscopy (Class I, scale bar = 2.0 μm). (C) Mild mesangial expansion (Class IIa). (D) Severe mesangial expansion (Class IIb). (E) Kimmelstiel–Wilson lesion (Class III). (F) Global glomerulosclerosis (Class IV).

All renal biopsy was performed with the consent of each patient. Tissue was obtained by needle biopsy, and the specimens were routinely processed for light microscopy (LM), immunofluorescence (IF), and electron microscopy (EM) to detect the renal pathological alterations. Each specimen was examined by the same group of pathologists. All patients were categorized according to the pathologic classification of the Renal Pathology Society [23]. Several typical pathologic features of different classes were illustrated in Figure 2.

### 2.4. Statistical analysis

Statistical analysis was performed using SPSS software (version 22.0; SPSS Inc., Chicago, IL). For continuous variables, data were presented as the mean ± standard deviation or median with range. Differences between groups were assessed with the *t*-test for normally distributed data and the Mann–Whitney *U* test for not normally distributed. The categorical variables were compared using the chi-squared test and Wilcoxon rank sum test. The logistic regression analyses were performed to identify risk factors for odds of DR. Renal outcome was assessed using Kaplan–Meier survival analysis, and survival rates were compared with the log-rank test. The association between DR and renal outcome was calculated using Cox regression. In Cox model 1, hazard ratio (HR) was adjusted for age, gender, hypertension, cigarette smoking, and the duration of T2DM at the time of renal biopsy. In model 2, HR was adjusted for all of the above covariates plus HbA1c, hematuria, and serum creatinine. In model 3, HR was adjusted for the clinical variables in model 2 and other renal pathological findings, such as the glomerular class and interstitial inflammation score. A two-tailed *p* < .05 was considered statistically significant.

## 3. Results

### 3.1. Clinical and pathologic characteristics of DN patients with and without DR in the cross-section study

The cross-section study included 250 patients, 172 were male (68.8%) and 78 were female (31.2%). At the time of renal biopsy, the mean age was 52.56 ± 8.68 years. The median DM duration was 78 months (range, 0–360 months). The mean baseline serum creatinine level was 1.57 ± 0.92 mg/dl, mean e-GFR was 68.46 ± 34.53 mL/min/1.73 m^2^, and median 24-h proteinuria level was 4.51 g/day (range, 0.04–27.00 g/day).

The DN group accounted for 52% (130/250), and the DN + DR accounted for 48% (120/250). Compared with the DN group, patients in DN + DR group were more likely to have longer duration of T2DM, higher levels of serum creatinine and proteinuria, lower levels of hemoglobin, increased proportion of hematuria, and decreased e-GFR than participants without DR (*p* < .05), as shown in Table 1. No differences in the age, gender distribution, incidence of hypertension, blood urea nitrogen (BUN), body mass index (BMI), fasting blood sugar (FBS), uric acid (UA), high density lipoprotein (HDL), low density lipoprotein (LDL), triglyceride, total cholesterol, or HbA1c level were observed in the two groups.

**Table 1.** Baseline demographics in the cross-section and cohort studies.

	Cross-section study					Cohort study		
Parameter	All (*n* = 250)	DN (*n* = 130)	DN + DR (*n* = 120)	*p* value*	All (*n* = 141)	DN (*n* = 68)	DN + DR (*n* = 73)	*p* value*
Men (%)	172 (68.8%)	90 (69.2%)	82 (68.3%)	.878	97 (68.8%)	48 (70.6%)	49 (67.1%)	.657
Age (years)	52.56 ± 8.68	53.35 ± 9.21	51.71 ± 8.03	.135	52.63 ± 8.19	53.12 ± 8.72	52.18 ± 7.71	.811
Cigarette smoking (%)	115 (46.4%)	59 (45.7%)	56 (47.1%)	.835	64 (45.4%)	28 (41.2%)	36 (49.3%)	.332
Hypertension (%)	212 (84.8%)	106 (81.5%)	106 (88.3%)	.135	119 (84.4%)	56 (82.4%)	63 (86.3%)	.519
Duration of diabetes (months)	78 (0–360)	60 (0–264)	96 (0–360)	.01	72 (0–360)	60 (0–240)	108 (0–360)	.002
Body mass index (kg/m^2^)	25.41 ± 4.16	25.28 ± 3.86	25.51 ± 4.40	.951	25.3 ± 4.68	25.04 ± 4.43	25.64 ± 4.91	.865
SBP (mmHg)	145.99 ± 24.66	144.04 ± 22.67	148.11 ± 26.58	.344	148.28 ± 23.74	146.87 ± 22.71	149.60 ± 24.75	.492
DBP (mmHg)	86.06 ± 13.66	86.08 ± 14.23	86.03 ± 13.08	.90	86.65 ± 13.19	87.38 ± 13.95	85.96 ± 12.50	.521
Hematuria (%)	162 (65.6%)	76 (59.4%)	86 (72.3%)	.033	93 (66%)	40 (59.7%)	53 (73.6%)	.082
24-h proteinuria (g/day)	4.51 (0.04–27)	3.81 (0.04–27.00)	5.255 (0.28–21.42)	.047	4.59 (0.04–22.5)	3.99 (0.04–22.5)	5.8 (0.28–19.35)	.021
BUN (mmol/L)	9.22 ± 5.08	8.73 ± 4.13	9.74 ± 5.91	.116	9.60 ± 4.01	9.35 ± 4.14	9.83 ± 3.91	.293
Serum creatinine (mg/dl)	1.57 ± 0.92	1.49 ± 1.01	1.67 ± 0.81	.001	1.73 ± 0.96	1.63 ± 1.05	1.82 ± 0.85	.016
e-GFR (mL/min/1.73 m^2^)	68.46 ± 34.53	75.92 ± 37.57	60.38 ± 28.93	<.001	63.41 ± 34.05	72.31 ± 38.55	55.13 ± 26.95	.006
Uric acid (mmol/L)	377.44 ± 77.26	378.07 ± 83.57	376.75 ± 70.12	.894	384.57 ± 78.82	391.64 ± 91.78	377.98 ± 64.41	.533
Fasting blood glucose (mmol/L)	8.19 ± 4.48	8.34 ± 4.09	8.01 ± 4.88	.579	8.36 ± 4.84	8.57 ± 4.64	8.17 ± 5.03	.138
Glycosylated hemoglobin (%)	7.49 ± 1.94	7.52 ± 1.76	7.45 ± 2.10	.387	7.24 ± 1.85	7.17 ± 1.48	7.29 ± 2.14	.732
HDL cholesterol (mmol/L)	1.42 ± 0.55	1.38 ± 0.55	1.45 ± 0.54	.362	1.42 ± 0.53	1.34 ± 0.45	1.49 ± 0.58	.087
LDL cholesterol (mmol/L)	3.27 ± 1.55	3.19 ± 1.45	3.34 ± 1.64	.456	3.27 ± 1.42	3.01 ± 1.07	3.52 ± 1.65	.07
Triglyceride (mmol/L)	2.24 ± 1.69	2.41 ± 1.93	2.05 ± 1.36	.098	2.17 ± 1.50	2.39 ± 1.77	1.95 ± 1.16	.177
Total cholesterol (mmol/L)	5.62 ± 2.11	5.63 ± 2.22	5.59 ± 1.98	.909	5.49 ± 1.71	5.19 ± 1.33	5.77 ± 1.97	.108
Hemoglobin (g/L)	119.18 ± 26.87	126.23 ± 26.62	111.67 ± 25.14	<.001	115.7 ± 24.72	124.57 ± 25.68	107.56 ± 20.86	<.001

BUN: blood urea nitrogen; DBP: diastolic blood pressure; e-GFR: estimated glomerular filtration rate; HDL: high density lipoprotein; LDL: low density lipoprotein; SBP: systolic blood pressure.

Data are presented as the mean ± standard, the median with range or counts and percentages.

*A two-tailed *p* < .05 was considered statistically significant.

Pathologic characteristics in DN patients with or without DR are shown in Table 2. According to the glomerular classifications of the Renal Pathology Society in 2010 [23], 2 patients with DR (12.5%) were in class I, 14 (28%) in class IIa, 12 (57.1%) in class IIb, 76 (60.8%) in class III, and 16 (42.1%) in class IV. Interstitial fibrosis and tubular atrophy (IFTA) of scores 0, 1, 2 and 3 were observed in 2 (15.4%), 57 (47.1%), 50 (53.2%), and 11 (50%) patients with DR, respectively. Interstitial inflammation of scores 0, 1 and 2 was observed in 4 (16.7%), 91 (51.1%) and 25 (52.1%) patients with DR, respectively. Arteriolar hyalinosis at scores of 0, 1 and 2 was observed in 18 (46.2%), 45 (45.0%), and 57 (51.4%) patients with DR, respectively. The Wilcoxon rank sum test demonstrated that the patients in DN + DR group had more serious glomerular lesions and interstitial inflammation (*p* < .05). However, there is no difference in IFTA and arteriolar hyalinosis scores. The chi-squared test indicated that both of the glomerular class IIb and III had a higher prevalence of DR than class IIa + I, and the interstitial inflammation of scores 1 and 2 had a higher prevalence of DR than the group with score 0 (*p* < .05). However, no difference about the percentage of DR was observed in the class IV versus class IIa + I.

**Table 2.** Pathological findings according to diabetic retinopathy.

	Diabetic retinopathy			
Pathological lesions	Absent (*n* = 130)	Present (*n* = 120)	Prevalence	*p* value*
Glomerular class				.003
I	14 (10.8%)	2 (1.7%)	12.5%	
IIa	36 (27.7%)	14 (11.7%)	28%	
IIb	9 (6.9%)	12 (10.0%)	57.1%£	
III	49 (37.7%)	76 (63.3%)	60.8%#	
IV	22 (16.9%)	16 (13.3%)	42.1%	
IFTA				.091
0	11 (8.5%)	2 (1.7%)	15.4%	
1	64 (49.2%)	57 (47.5%)	47.1%	
2	44 (33.8%)	50 (41.7%)	53.2%	
3	11 (8.5%)	11 (9.2%)	50.0%	
Interstitial inflammation				.031
0	20 (15.4%)	4 (3.3%)	16.7%	
1	87 (66.9%)	91 (75.8%)	51.1%§	
2	23 (17.7%)	25 (20.8%)	52.1%§	
Arteriolar hyalinosis				.399
0	21 (16.2%)	18 (15.0%)	46.2%	
1	55 (42.3%)	45 (37.5%)	45.0%	
2	54 (41.5%)	57 (47.5%)	51.4%	

Prevalence indicted the percentage of the patients with DR in the total patients of each row.

IFTA: interstitial fibrosis and tubular atrophy.

*Wilcoxon Rank sum test. A two-tailed *p* < .05 was considered statistically significant.

Chi-squared test: £*p* < .05 versus class IIa and I. #*p* < .001 versus class IIa and I. §*p* < .05 versus score 0.

### 3.2. Risk factors for the odds of DR

**Figure 3.**
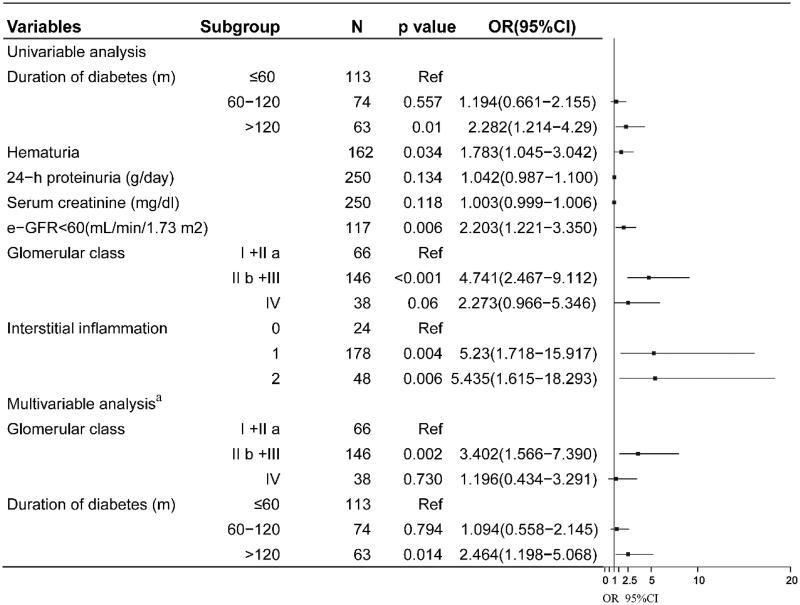
Risk factors for DR identified by multivariate binary logistic regression analysis. Ref indicated reference group. **^a^**Adjusting for baseline proteinuria, hematuria, e-GFR, and interstitial inflammation.

A univariate logistic regression analysis was performed to identify potential risk factors for the odds of DR. As shown in Figure 3, patients with a T2DM history >10 years had a higher prevalence of DR (OR, 95%CI; 2.282 (1.214–4.29); *p* = .01), and diabetic patients with hematuria had a higher risk for DR (OR, 95%CI; 1.783 (1.045–3.042); *p* = .034). In addition, the glomerular lesions (class IIb + III versus IIa + I) were significantly associated with DR (OR, 95%CI; 4.741 (2.467–9.112); *p* < .001), but the class IV did not significantly influence the odds of DR (*p* = .06). What is more, the participants with a serious interstitial inflammation (score 1 and 2) had higher risk for the prevalence of DR (OR, 95%CI; 5.23 (1.718–15.917); *p* = .004; and 5.435 (1.615–18.293); *p* = .006, respectively). After adjusting for age, sex, baseline proteinuria, hematuria, e-GFR, and interstitial inflammation, the results showed that the severity of glomerular lesions (class IIb + III) and DM history >10 years remained an independent risk factors (OR, 95%CI; 3.402 (1.566–7.390) and 2.464 (1.198–5.068), respectively) for odds of DR.

### 3.3. Clinical characteristics of patients in the cohort study

The 141 patients were also divided into DN + DR and DN groups. The DN group accounted for 48% (68/141), and the DN + DR accounted for 52% (73/141). Compared with the DN group, patients in DN + DR group were more likely to have longer duration of T2DM, higher levels of serum creatinine and proteinuria, and decreased e-GFR (*p* < .05), as shown in Table 1. The median follow-up period was 19 months. 59 patients (41.8%), 38 in the DN + DR group and 21 in the DN group, progressed to ESRD and a total of 22 patients died during follow-up.

### 3.4. DR and renal outcomes

**Figure 4.**
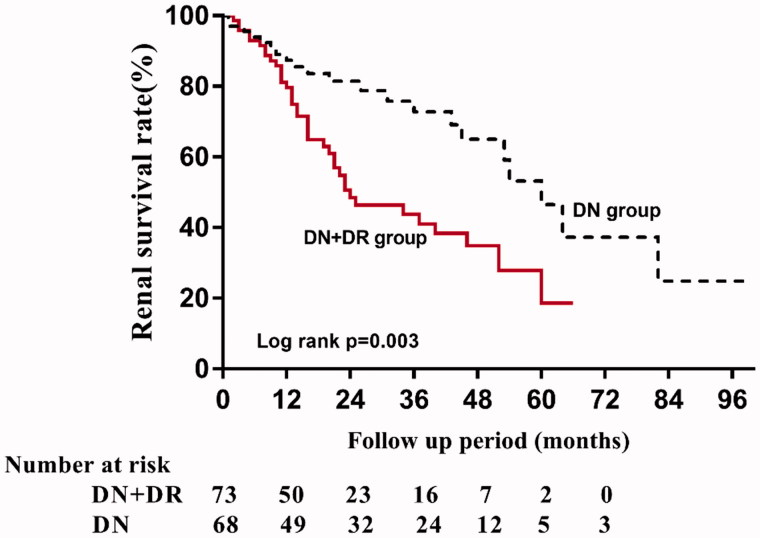
Kaplan–Meier curves of renal survival rate in DN patients with or without DR.

**Figure 5.**
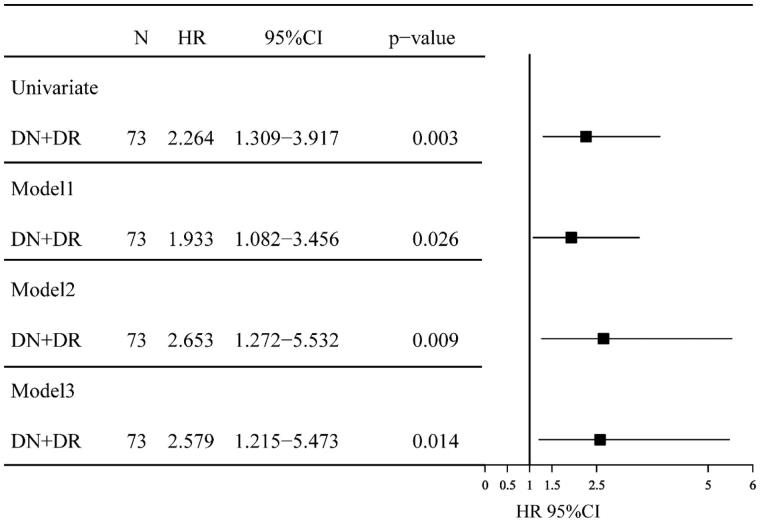
HRs of progression to ESRD for the patients with DN + DR versus DN only in the cohort study using univariate/multivariate COX hazard analysis. CI: confidence interval; HR: hazard ratio; Model 1: adjusted for age, gender, hypertension, cigarette smoking and the duration of T2DM at the time of renal biopsy; Model 2: adjusted for all of the above covariates plus HbA1c, hematuria and serum creatinine; Model 3: adjusted for the clinical variables in model 2 and other renal pathological findings, such as the glomerular class and interstitial inflammation score.

Survival curves of the end point are shown in Figure 4, the Kaplan–Meier survival analysis showed that patients with and without DR had five-year renal survival rates of 18.6% and 46.6%, respectively. There was a significant difference of renal survival among the two groups (*p* = .003). The univariate Cox analysis showed that patients with DR had a lower renal survival rate than patients without (hazard ration (HR), 95%IC; 2.264 (1.309–3.917), *p* = .003). The adjusted HRs of DR for renal survival was shown in Figure 5. In model 1, the HR for DN + DR group was significantly higher compared with DN group, being 1.933 (95% CI: 1.082–3.456, *p* = .026). In model 2, the HR for DN + DR group were also significantly higher compared with DN group, being 2.653 (95% CI: 1.272–5.532, *p* = .009). In model 3, the HR for DN + DR were adjusted for clinical variables in model 2 and other renal pathological findings, and was 2.579 (95%CI: 1.215–5.473, *p* = .014) compared with DN group.

## 4. Discussion

The cross-section study revealed that the prevalence of DR in patients with biopsy-proven DN was 48%, and these patients had longer duration of T2DM, poorer renal function, more serious glomerular lesions, and interstitial inflammation (*p* < .05) than the DN group. Furthermore, the severity of glomerular lesions and duration of DM were significantly associated with the odds of DR, independent of clinical features. Interstitial inflammation influenced the odds of DR using a univariate logistic regression analysis but failed to be an independent risk factor. In the cohort study, multivariate COX analysis demonstrated that the DR was significantly associated with renal outcomes when adjusting for important clinical variables and pathological changes (*p* < .05).

These findings offer further insights into the pathophysiology of renal lesions and the odds of DR. Klein et al. [24] and Kofoed-Enevoldsen et al. [25] reported that similar molecular pathways appear to govern the development of diabetic renal and retinal microvascular injury. This speculation stems from higher coincidence rates of DN and DR; that is, patients with DN may have already developed DR and patients with DR are vulnerable to develop DN. These previous studies demonstrated a significant link between DR and DN, and this present report also suggested that the presence of one preexisting microvascular complication (DR or DN) may contribute to the development of another which made that conclusion more convincing in the light of the diagnosis of DN based on renal biopsy in our research.

Renal pathology patterns related with DR in T2DM was described in the 1990s by Schwartz et al. [26] and Olsen et al. [27]. They pinpointed that the correlation between DR and the Kimmelstiel–Wilson (KW) lesion but not the mesangial sclerosis (MS) lesion suggested that the KW and MS lesions were most likely caused by different pathogenic mechanisms. Findings presented in this investigation showed that DR was associated with the glomerular class IIb + III (severe mesangial expansion and nodular sclerosis) but not the class IV (global glomerulosclerosis), which was largely in accordance with the previous study. The difference might result from the different pathologic classification. KW lesions are often found in combination with mesangial expansion. The occurrence of KW lesions is widely considered transitional from an early or moderately advanced stage to a progressively more advanced stage of disease [28,29]. And the glomerulosclerosis in DN was regarded as the end point of multifactorial mechanisms which through stages of mesangial expansion and development of KW lesions finally results in glomerulosclerosis [30]. Whether the nodular sclerosis and global glomerulosclerosis were induced by different mechanisms awaits further investigation.

What is more, this study suggested that renal interstitial inflammation also influenced the odds of DR. Over the past decades, there have been important advances in understanding the pathogenesis of DN and DR, with particular focus on oxidative stress and inflammatory status. Oxidative stress plays an important role in the development of diabetic vascular complications, including DN and DR. Recently, Jinkui Yang et al. [31] have reported that urinary haptoglobin, which is specific for eye damage, is a putative clinical biomarker for predicting kidney damage related to diabetes. And the haptoglobin polymorphism could contribute to the prevalence and clinical evolution of many inflammatory diseases, including T2DM [32] and atherosclerosis [33]. The mechanism of these effects may be a phenotype-dependent modulation of oxidative stress. In addition, in T2DM, the development of nephropathy is associated with the activation of CD8 + T cells and with the increase of interleukin-6 (IL-6), NLRP3 inflammasome, and TNFR1/2 (Tumor necrosis factor receptors 1 and 2) [34–37]. And the retinal inflammation also plays a critical role in the pathogenesis of DR, such as TXNIP/NLRP3 inflammasome [37] and IL-6 [38]. Together with diabetes-induced AGEs formation and impaired endogenous anti-inflammatory pathways lead to chronic inflammatory reactions in the retina by persistently inducing expression of inflammatory cytokines, chemokines, and recruiting leukocytes [39]. Different studies have shown that DR as well as DN is a multi- factorial disease involving multiple pathways, including aldose reductase pathway, oxidative stress, activation of PKC, complement activation, and formation of AGEs [39–42]. In the current study, we also addressed that interstitial inflammation is associated with the occurrence of DR. However, the functional roles of these inflammatory cytokines in regulating the interplay between DR and DN have not been defined. More prospected studies are needed if we are to know the exact mechanism of how these diabetic microvascular diseases correlate.

Our present study also revealed strong associations between the presence of DR and the renal outcomes in T2DM patients. These findings suggested a potential ‘common pathway’ between the DR and renal dysfunction in that findings of retinopathy may be representative of systemic microvascular damage secondary to diabetes that lead to both progressive renal dysfunction and breakdown of the blood vessel–retinal tissue barrier [16]. Furthermore, the presence of DR may identify individuals with predisposing conditions, who may be at increased risk for DN or more serious renal lesions caused by similar microvascular damage. Although several candidate genes associated with susceptibility to DR and DN had been reported, the specific mechanisms promoting the occurrence of DR and DN have not been defined so far [43–45]. By extension, these findings potentially could identify individuals in need of aggressive treatment with agents (such as ACE inhibitors) designed to minimize microvascular complications.

With these implications aside, several limitations of this study deserved comment. Firstly, it was a retrospective study in a single center, which led to sampling bias and a limited sample size, and selection bias was inevitable in any biopsy-based study; secondly, given the study design, we could not prove the causality between DR and progression of DN; thirdly, diabetic retinopathy examination of many patients was performed just by ophthalmoscopy after mydriasis at baseline and the stages of DR were not classified, whether the patients in DN group will develop DR in the future is unclear; fourthly, the role of some unmeasured confounding factors that could have possibly influenced the observed association cannot be entirely ruled out; moreover, we did not evaluate the therapeutic interventions during follow-up, which may have potential impact on renal prognosis; Finally, the T2DM patients in this study were all from southwest of China and most of them were with heavy proteinuria, so the results might be different with the study recruiting T1DM patients, or T2DM patients with different geographic and genetic background, especially with normoalbuminuria or microalbuminuria.

In conclusion, this study showed the strong associations between DR and DN in patients with T2DM, and DR was an independent risk factor for the renal outcomes in patients with DN. Nevertheless, current treatments for both diabetic nephropathy and retinopathy have not been completely effective in delaying or halting the development of diseases, suggesting that further understanding of the molecular mechanisms underlying the pathogenesis of both diabetic nephropathy and retinopathy are necessary. For now, in order to have a positive impact on T2DM patients’ quality of life, diabetic microvascular complications (DR and DN) should be early identified in those patients with T2DM and be treated together.

## Disclosure statement

No potential conflict of interest was reported by the authors.
